# Amplicon-Based High-Throughput Sequencing Method for Genotypic Characterization of Norovirus in Oysters

**DOI:** 10.1128/aem.02165-22

**Published:** 2023-04-18

**Authors:** Amy H. Fitzpatrick, Agnieszka Rupnik, Helen O’Shea, Fiona Crispie, Paul D. Cotter, Sinéad Keaveney

**Affiliations:** a Department of Food Biosciences, Teagasc Food Research Centre, Fermoy, Ireland; b Department of Shellfish Microbiology, Marine Institute, Oranmore, Ireland; c Department of Biological Sciences, Munster Technological University, Cork, Ireland; Centers for Disease Control and Prevention

**Keywords:** environmental virology, *Caliciviridae*, Illumina, DNA polymerase, reverse transcriptase, DNA sequencing, calicivirus, environmental microbiology, food safety, virology

## Abstract

Norovirus is a highly diverse RNA virus often implicated in foodborne outbreaks, particularly those associated with shellfish. Shellfish are filter feeders, and when harvested in bays exposed to wastewater overflow or storm overflows, they can harbor various pathogens, including human-pathogenic viruses. The application of Sanger or amplicon-based high-throughput sequencing (HTS) technologies to identify human pathogens in shellfish faces two main challenges: (i) distinguishing multiple genotypes/variants in a single sample and (ii) low concentrations of norovirus RNA. Here, we assessed the performance of a novel norovirus capsid amplicon HTS method. We generated a panel of spiked oysters containing various norovirus concentrations with different genotypic compositions. Several DNA polymerases and reverse transcriptases (RTs) were compared, and performance was evaluated based on (i) the number of reads passing quality filters per sample, (ii) the number of correct genotypes identified, and (iii) the sequence identity of outputs compared to Sanger-derived sequences. A combination of the reverse transcriptase LunaScript and the DNA polymerase AmpliTaq Gold provided the best results. The method was then employed, and compared with Sanger sequencing, to characterize norovirus populations in naturally contaminated oysters.

**IMPORTANCE** While foodborne outbreaks account for approximately 14% of norovirus cases (L. Verhoef, J. Hewitt, L. Barclay, S. Ahmed, R. Lake, A. J. Hall, B. Lopman, A. Kroneman, H. Vennema, J. Vinjé, and M. Koopmans, Emerg Infect Dis 21:592–599, 2015), we do not have standardized high-throughput sequencing methods for genotypic characterization in foodstuffs. Here, we present an optimized amplicon high-throughput sequencing method for the genotypic characterization of norovirus in oysters. This method can accurately detect and characterize norovirus at concentrations found in oysters grown in production areas impacted by human wastewater discharges. It will permit the investigation of norovirus genetic diversity in complex matrices and contribute to ongoing surveillance of norovirus in the environment.

## INTRODUCTION

In norovirus outbreaks associated with the consumption of shellfish, clinical and shellfish samples collected during the outbreak investigation are often subjected to nucleic acid Sanger sequencing for source attribution. The application of Sanger sequencing in this scenario is cumbersome in shellfish due to the need for the cloning of PCR isolates to capture the genetic diversity in a single sample (1–3) and generally allows only low-throughput analysis. High-throughput sequencing (HTS) permits low-cost sequencing, yields high-throughput data, and captures considerable nucleotide diversity, as demonstrated with 16S rRNA amplicon-based HTS analysis of bacteriomes. In contrast to Sanger sequencing, HTS technologies can also resolve multiple sequences per amplicon without cloning of isolates in environmental samples.

A few studies have applied HTS-based methods for the detection and characterization of norovirus in complex matrices, such as food and wastewater (4–16). These studies notwithstanding, the performance of the various methods, whether it be shotgun metagenomics, capture probe hybridization, long-read sequencing, or amplicon-based HTS, has varied. While shotgun metagenomics permits a less biased approach to sequencing, sequencing depth is often insufficient to characterize the norovirus present due to the presence of nucleic acid from other sources, even after rRNA removal or poly(A) tail enrichment (7, 8, 11, 12, 14). Capture probe hybridization can enrich viral sequences, which could be helpful in challenging matrices. However, current market options are expensive and do not necessarily enrich the regions used for dual genotyping of norovirus, as panels are designed to broadly target viruses rather than targeting specific viral families (7–9, 16). Long-read sequencing methods, such as those provided by PacBio and Oxford Nanopore Technologies (ONT), also face challenges in obtaining sufficient sequencing depth in complex matrices. Indeed, their outputs are typically lower than those from short-read platforms. ONT combined with adaptive sampling (a target or host enrichment or depletion algorithm) has had limited success in food samples (7), while, to date, long-read ONT sequencing of norovirus amplicons has not been successful (16). Despite these challenges, several recent studies have demonstrated the capability of various amplicon HTS assays to be used for the genotypic characterization of norovirus in shellfish (5, 16), with similar success observed in berry samples (6). However, these studies focused on application rather than optimization and did not confirm HTS results with the gold standard Sanger methods. Due to the high degree of underreporting of norovirus cases, particularly nonnosocomial cases in healthy populations (17, 18), samples tend to come from foodborne outbreaks requiring source attribution or chronic nosocomial cases. This skews our understanding of norovirus genotypes circulating in local populations and will limit effective vaccine production and management of clinical cases (19). Given the necessity of capturing the norovirus sequences that permit genotypic characterization in complex samples with low concentrations of viral RNA, this study focused on optimising amplicon-based HTS methods.

Despite its potential value, amplicon HTS can introduce bias at multiple steps in the process, most notably RNA extraction and amplification of the target DNA. Bias during PCR cycling is impacted by the choice of primers, in that they are designed to target a conserved area of a chosen genome (20, 21). Currently, norovirus taxonomic assignment relies on dual genotyping based on the RNA-dependent RNA polymerase (RdRp) and VP1 gene (22). However, historically genotypic characterization has focused on the VP1, also known as the capsid (23, 24). As a single-stranded RNA (ssRNA) virus, norovirus has a high mutation rate, estimated to be 5.40 × 10^−3^ to 2.23 × 10^−4^ nucleotide substitution/site/year for the VP1-coding region (25). Moreover, noroviruses are genetically diverse, with a relatively low shared nucleotide identity of approximately 63% across commonly sequenced regions, such as region C and the breakpoint of the RdRp and VP1 (22). Thus, designing suitable primers to capture norovirus’s existing and potential diversity is challenging. Various primers have been used for molecular characterization, with most reference laboratories targeting the most conserved region of the genome (open reading fram 1 [ORF1]-ORF2 junction) and using degenerate primers that can tolerate sequence mismatches (24, 26). The regions typically targeted for molecular characterization result in amplicons ranging from 113 bp to 587 bp in length (24). The present study generated amplicons using primers targeting region C, which yielded a 340-bp amplicon suitable for 300-bp paired-end sequencing. These primers are highly degenerate and can capture a broad range of norovirus genotypes.

The objective of this study was to establish a capsid amplicon-based HTS method for norovirus genotyping in shellfish. Criteria for a successful assay were as follows: (i) accurate characterization of genotypes in samples with concentrations of norovirus RNA typically observed in outbreaks, (ii) accurate characterization of multiple genotypes, and (iii) performance superior to that of Sanger sequencing for application in surveillance-based studies. Reverse transcriptases (RTs) and DNA polymerases are known to impact HTS outputs from quality to classification accuracy (27–30); therefore, selected enzyme combinations were compared in spiked and naturally contaminated samples.

The optimized amplicon-based HTS method and traditional Sanger sequencing method were successfully applied to a panel of naturally contaminated oysters, with a strong agreement between conventional and novel techniques.

## RESULTS

The method was optimized with clinical samples and either oysters spiked with clinical material or naturally contaminated samples. By sequencing previously characterized clinical and spiked samples, method performance could be assessed in terms of its sensitivity and specificity.

### Characterization of clinical positive-control samples.

To facilitate the subsequent assessment of the accuracy of HTS-based approaches, it was necessary first to obtain clinical samples containing specific norovirus genotypes. RT quantitative PCR (RT-qPCR) and Sanger sequencing were used to characterize these clinical samples (Table 1), which were positive for one genotype per sample and contained a high concentration of norovirus RNA. These samples were subsequently used to spike oyster samples.

**TABLE 1:** Sanger sequencing results for clinical samples used in the proof-of-concept library and spiking experiments^*a*^

Sample ID	NoV gc/μL		Genotype in NoroNet
	GI	GII	
FM011		1.03 × 10^4^	GII.3
FM012		1.67 × 10^3^	GII.2
FM018		1.18 × 10^5^	GII.4 Sydney 2012
FM022	5.47^3^		GI.4
FM023		3.23 × 10^6^	GII.4
FM026	3.26^2^		GI.9

a NoV, norovirus. Genotypic characterization of Sanger sequences from each sample was performed using NoroNet.

### Impact of enzyme combinations on genotype detection by HTS.

Three reverse transcriptase and DNA polymerase combinations were applied to clinical and spiked shellfish samples. Table 2 provides the genotypes detected in the various sequencing experiments (experiments 1 to 3) by the enzyme combinations. Samples in experiment 1 included the clinical samples (Table 1) and spiked shellfish samples that were prepared using material from the clinical samples at various concentrations and combinations. An additional spiked shellfish sample was sourced for a comprehensive RT and DNA polymerase comparison in experiment 2. The optimized method was applied to a panel of naturally contaminated samples in experiment 3, using LunaScript with AmpliTaq Gold.

**TABLE 2:** Genotypes detected using amplicon HTS across all sample types prepared with selected RT and DNA polymerases^*a*^

Enzyme combination	Expt	Genotypes in NoroNet
SuperScript II–AmpliTaq Gold	1	GI.4, GI.9, GII.2, GII.3, GII.4 New Orleans 2009, GII.4 Sydney 2012
SuperScript II–Kapa HiFi	1	GI.4, GII.2, GII.3, GII.4 New Orleans 2009, GII.4 Sydney_2012
SuperScript II–Kapa Robust	1	GI.4, GI.9, GII.2, GII.3, GII.4 New Orleans 2009, GII.4 Sydney 2012
SuperScript II–AmpliTaq Gold	1	GI.4, GI.9, GII.2, GII.3, GII.4, GII.4 Sydney 2012
SuperScript II–Kapa HiFi	1	GI.4, GI.9, GII.2, GII.3, GII.4, GII.4 Sydney 2012
SuperScript II–Kapa Robust	1	GI.4, GI.9, GII.2, GII.3, GII.4, GII.4 Sydney 2012
LunaScript–AmpliTaq Gold	2	GI.1, GI.3, GI.7, GII.6
LunaScript–Kapa HiFi	2	GI.1, GI.3, GI.7, GII.6
LunaScript–Kapa Robust	2	GI.3, GI.7, GII.4 Sydney 2012, GII.6
SuperScript II–AmpliTaq Gold	2	GI.3, GI.7, GII.2, GII.6
SuperScript II–Kapa HiFi	2	GI.3, GI.7, GII.2, GII.6
SuperScript II–Kapa Robust	2	GI.3, GI.7, GII.2, GII.6
SuperScript IV–AmpliTaq Gold	2	GI.3, GI.7, GII.6, GII.7
SuperScript IV–Kapa HiFi	2	GI.3, GI.7, GII.6, GII.7
SuperScript IV–Kapa Robust	2	GI.1, GI.3, GI.7, GII.2, GII.6, GII.7
LunaScript–AmpliTaq Gold	3	GI.3, GI.7, GI.9, GII.13, GII.14, GII.4 Sydney 2012, GII.6, GII.7

a Genotypic characterization of HTS OTUs was performed using NoroNet.

### Enzyme combination impacts the quality of high-throughput sequencing reads.

Selected RTs and DNA polymerases were compared to determine whether method optimization could improve the quality of sequences obtained. The Moloney murine leukemia virus (MMLV)-derived RT SuperScript II and SuperScript IV were evaluated alongside an *in silico*-designed RT, LunaScript. The polymerases were selected because AmpliTaq Gold is widely used for RNA virus sequencing; Kapa HiFi has a low error rate and is recommended for use in 16S amplicon HTS protocols on Illumina platforms. In contrast, KAPA2G Robust has a greater tolerance for PCR inhibitors and is recommended for use with challenging samples.

**FIG 1:**
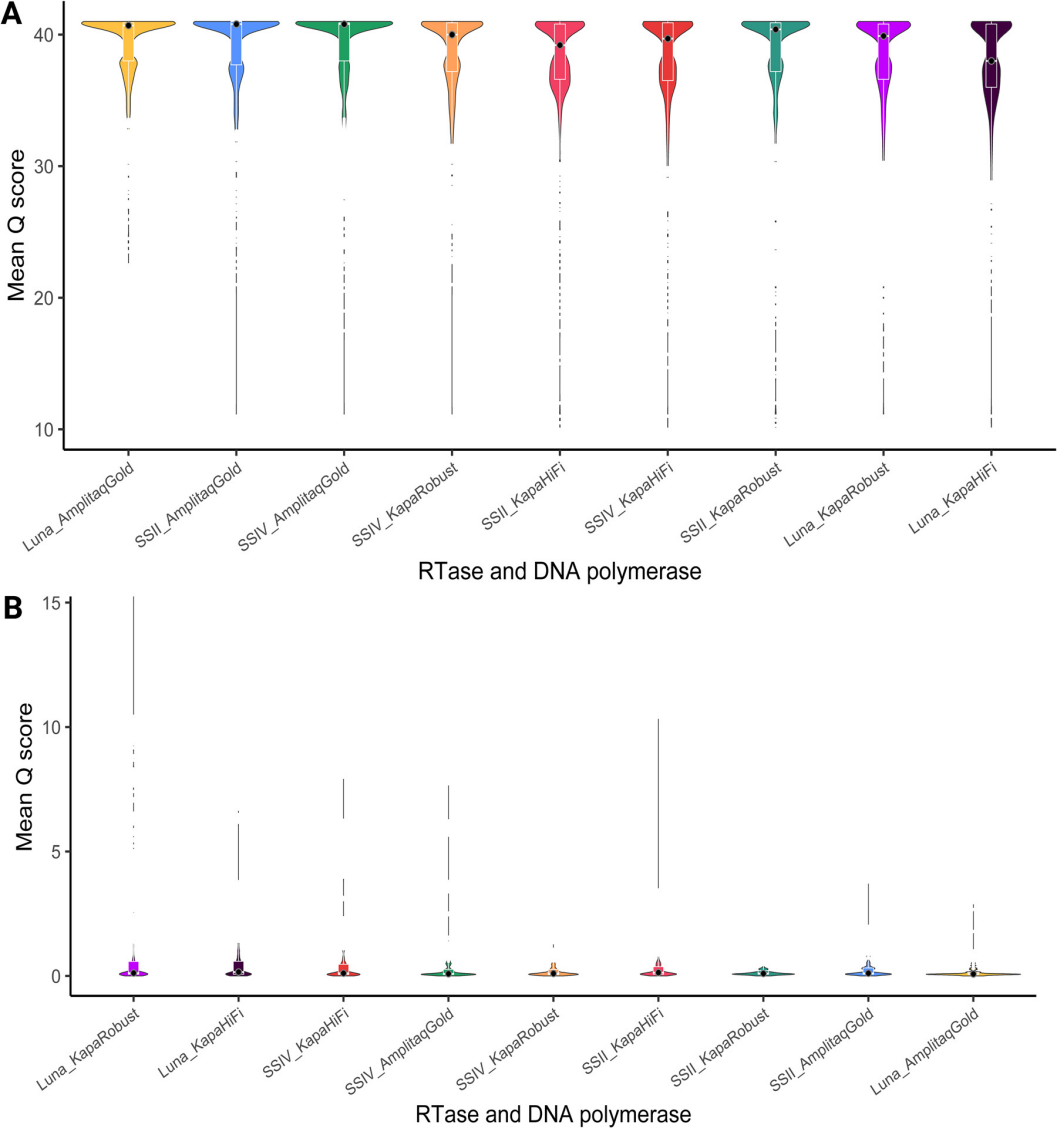
(A) Violin plots with internal box plots of the mean Phred score (*Q*) obtained in spiked shellfish samples and naturally contaminated oysters. RT and DNA polymerase combinations are ordered from highest mean Phred score to lowest (left to right). (B) Violin plots with internal box plots of the mean expected errors (EE). RT and DNA polymerase combinations are ordered from the highest mean expected error score to the lowest (left to right).

Results were compared based on Phred quality scores, used to indicate the base quality in DNA sequencing and expected errors, which are the sum of error probabilities over the length of the read. Mean Phred scores obtained (spiked shellfish) were significantly different when assessed using a Kruskal-Wallis test (Fig. 1A) (*P* = 2.96 × 10^−12^; chi-squared = 425.56). RT and DNA polymerases are arranged from highest to lowest median Phred score, with LunaScript–AmpliTaq Gold generating sequences with the highest Phred scores and LunaScript–Kapa HiFi generating sequences with the lowest Phred scores. Expected errors (EE) were significantly different using a Kruskal-Wallis test (Fig. 1B) (*P* < 2.2 × 10^−16^; chi-squared = 1,971.1). LunaScript–AmpliTaq Gold generated sequences with the lowest mean EE, while LunaScript–Kapa Robust generated sequences with the highest mean EE.

**FIG 2:**
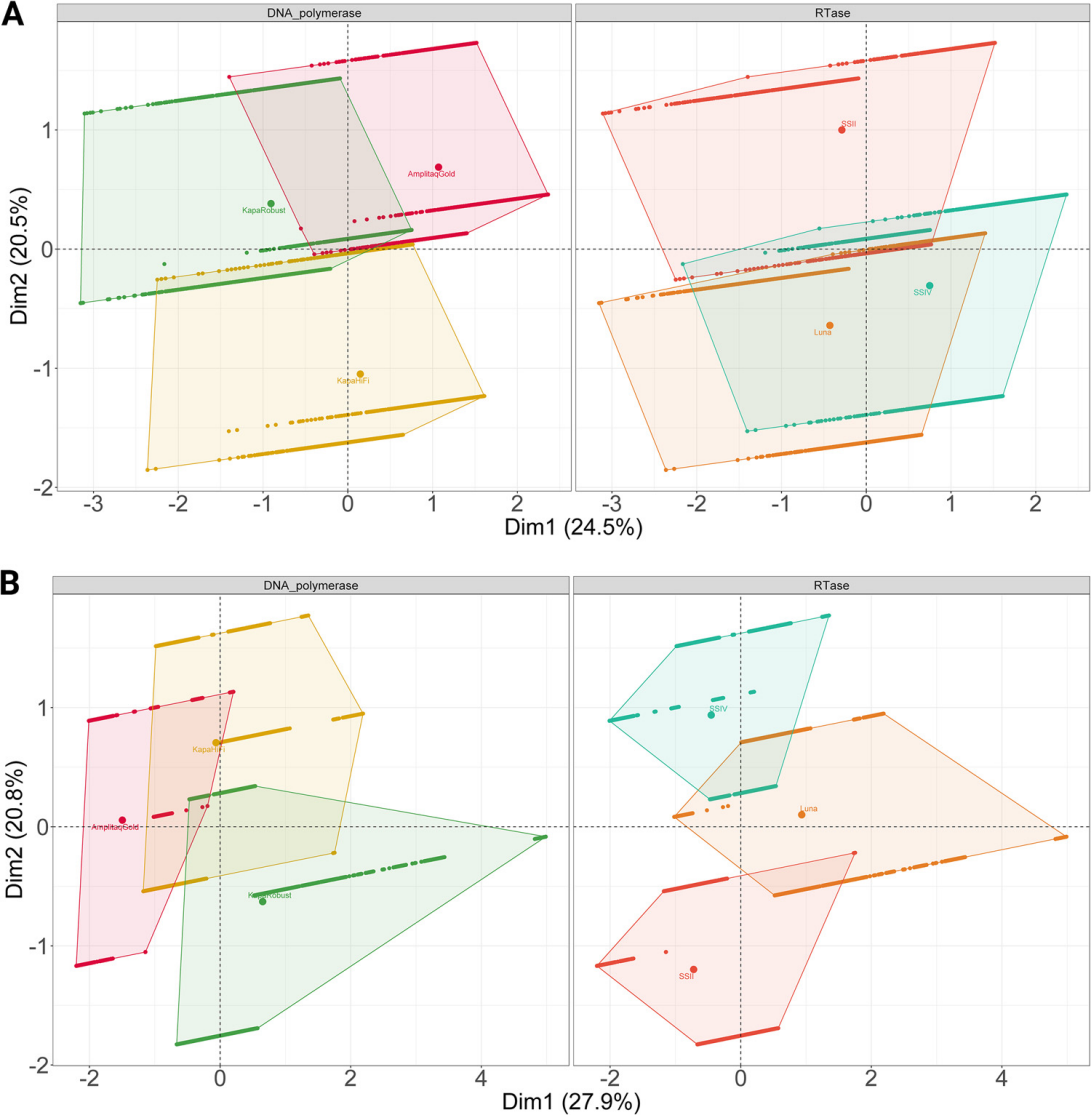
(A) FAMD biplot demonstrating the variance-maximizing distribution patterns of the total mean Phred scores in the map space and their clustering patterns based on DNA polymerase and RT. (B) FAMD biplot for DNA polymerase demonstrates the variance-maximizing distribution patterns of the total mean expected errors in the map space and their clustering patterns based on DNA polymerase and RT.

A factor analysis of mixed data (FAMD) was performed to determine if the choice of RT or DNA polymerase contributed to the differences in performance in terms of quality (Fig. 1). A FAMD works as a principal-component analysis (PCA) for quantitative variables and a multiple-correspondence analysis (MCA) for qualitative variables, allowing us to understand the relationship between numeric outcomes such as Phred score and factors such as DNA polymerase. DNA polymerases contributed to 53% and 54% of the variation observed in dimensions 1 and 2, respectively (Fig. 2A); however, the overall cos2 value was low (<0.3). A low cos2 indicates that the principal component does not perfectly represent DNA polymerases; i.e., other factors contribute to the variance observed. RTs explained 22% and 49% of the variation observed in dimensions 1 and 2, respectively, though the cos2 value was low (<0.3).

In summary, DNA polymerase contributed to observed differences in mean Phred score to a greater extent than the RT. On the other hand, as shown in Fig. 2B, DNA polymerase returned values of 42% and 35% while RT returned 39% and 68% for dimensions 1 and 2, respectively. Cos2 values for both reagents were low, <0.3. Accordingly, mean EE was influenced by RT more than DNA polymerases. Therefore, RT choice has a greater impact on expected mean errors, while DNA polymerase has a greater effect on mean Phred scores.

### DNA polymerase impacts the relationship between input genomic material and the number of HTS reads obtained.

A Kendall rank correlation coefficient was used to compare the agreement between the concentration of DNA following library preparation or the number of genome copies (gc) of norovirus per gram as determined by RT-qPCR before the seminested PCR and the number of HTS reads passing quality control. There was a perfect agreement (>0.8) between the concentration of DNA following library preparation as quantified by fluorometric measures and the resulting reads passing quality control for SuperScript II AmpliTaq Gold shellfish samples spiked with a single norovirus genotype (Table 3) (31). These correlations were not statistically significant, likely owing to the small sample size. Oyster samples that were spiked with multiple genotypes and prepared with SuperScript II AmpliTaq Gold provided a perfect agreement (>0.8) between the concentration of DNA following library preparation as quantified by fluorometric measures and the resulting reads passing quality control at statistically significant levels (Table 3). Overall, there was a weaker agreement between gc of norovirus per gram as determined by RT-qPCR and reads passing quality control.

**TABLE 3:** Kendall correlation between concentrations of norovirus or of DNA and reads after QC with HTS reads in spiked oyster samples^*a*^

DNA polymerase^*b*^	No. of genotypes	Kendall correlation with reads after QC (*P*) for:		*n*
		gc/g of norovirus detected by RT-qPCR	ng/μL of DNA	
AmpliTaq Gold	Single	0.667 (0.33)	1.000 (0.08)	4
Kapa Robust	Single	0.000 (1.00)	0.000 (1.00)	4
AmpliTaq Gold	Multiple	−0.247 (0.24)	0.871 (<0.05)	6
Kapa HiFi	Multiple	0.871 (0.51)	−0.567 (<0.05)	6
Kapa Robust	Multiple	−0.141 (<0.05)	0.591 (<0.05)	6

a Kendall correlation between concentrations of norovirus detected by RT-qPCR prior to seminested PCR or of DNA following seminested PCR and library preparation and reads after quality control (QC) with HTS reads following QC in spiked shellfish samples.

b The RT was SuperScript II.

Ultimately, in naturally contaminated samples (*n* = 9), there was a moderate nonsignificant agreement of 0.481 (*P* = 0.10) and 0.389 (*P* = 0.18), respectively, between gc of norovirus per gram as determined by RT-qPCR and concentration of DNA following library preparation as quantified by fluorometric measures and the resulting reads passing quality control.

### Enzyme combination and technical triplicates can improve classification accuracy.

A confusion matrix was used to investigate further the differences in performance between reverse transcription and DNA polymerases. As can be seen in Table 4, all enzyme combinations performed well when used with spiked shellfish samples. When the mock communities were prepared individually, all enzyme combinations returned perfect scores, apart from instances where SuperScript II was applied in combination with Kapa HiFi. For this library, genotype GII.3 norovirus was not detected in mock community 8, present at 700 gc/g (single genotype). Libraries where a spiked sample was prepared in triplicate from seminested PCR with LunaScript or SuperScript IV provided perfect F scores, in contrast to samples prepared with SuperScript II, which did not provide perfect F scores. The spiked sample contained GI.3, GI.7, and GII.6. GI.7 was not detected in samples prepared with SuperScript II in combination with AmpliTaq Gold or Kapa Robust. When the PCR products for norovirus GI and GII were combined, a loss in sensitivity was observed, i.e., GI.3 and GI.7 were not detected (Table 5). Similar trends were observed using the Jaccard index (see Fig. S1 in the supplemental material).

**TABLE 4:** Performance measurement of the HTS amplicon method with selected RT and DNA polymerase combinations in spiked oyster samples^*a*^

RT–DNA polymerase combination	Technical replicates	Accuracy	Sensitivity	Specificity	Precision	Recall	F score
LunaScript–AmpliTaq Gold	Triplicate	1.00	1.00	1.00	1.00	1.00	1.00
LunaScript–Kapa HiFi	Triplicate	1.00	1.00	1.00	1.00	1.00	1.00
LunaScript–Kapa Robust	Triplicate	1.00	1.00	1.00	1.00	1.00	1.00
SuperScript II–AmpliTaq Gold	Individual	1.00	1.00	1.00	1.00	1.00	1.00
	Triplicate	0.86	0.83	1.00	1.00	0.83	0.91
SuperScript II–Kapa HiFi	Individual	0.96	0.95	1.00	1.00	0.95	0.98
	Triplicate	1.00	1.00	1.00	1.00	1.00	1.00
SuperScript II–Kapa Robust	Individual	1.00	1.00	1.00	1.00	1.00	1.00
	Triplicate	0.86	0.83	1.00	1.00	0.83	0.91
SuperScript–IV AmpliTaq Gold	Triplicate	1.00	1.00	1.00	1.00	1.00	1.00
SuperScript–IV Kapa HiFi	Triplicate	1.00	1.00	1.00	1.00	1.00	1.00
SuperScript–IV Kapa Robust	Triplicate	1.00	1.00	1.00	1.00	1.00	1.00

a Performance was assessed to genotype-level classification using a confusion matrix.

**TABLE 5:** Performance measurement of the HTS amplicon method with selected RT and DNA polymerase combinations performed on pooled GI and GII amplicons in spiked oyster samples^*a*^

RT–DNA polymerase combination	Accuracy	Sensitivity	Specificity	Precision	Recall	F score
LunaScript–Kapa HiFi	1.00	1.00	1.00	1.00	1.00	1.00
SuperScript II–Kapa HiFi	1.00	1.00	1.00	1.00	1.00	1.00
SuperScript IV–Kapa HiFi	1.00	1.00	1.00	1.00	1.00	1.00
LunaScript–Kapa Robust	1.00	1.00	1.00	1.00	1.00	1.00
SuperScript IV–AmpliTaq Gold	0.86	0.83	1.00	1.00	0.83	0.91
SuperScript II–Kapa Robust	0.86	0.83	1.00	1.00	0.83	0.91
SuperScript IV–Kapa Robust	0.86	0.83	1.00	1.00	0.83	0.91
LunaScript–AmpliTaq Gold	0.71	0.67	1.00	1.00	0.67	0.80
SuperScript II–AmpliTaq Gold	0.71	0.67	1.00	1.00	0.67	0.80

a Performance was assessed to genotype-level classification using a confusion matrix.

### Phylogenetic distance between expected and observed sequences was affected by DNA polymerase.

UniFrac is a distance matrix that measures the phylogenetic distance between sets of taxa in a phylogenetic tree. The distance is defined as the fraction of the branch length of the tree that leads to descendants from either one environment or the other, but not both. Unweighted UniFrac methods were used to compare the phylogenetic distance between sequences generated by SuperScript II RT and one of the following DNA polymerases: AmpliTaq Gold, Kapa Robust, and Kapa HiFi. It demonstrated that DNA polymerase and RT explained some variations in sequencing results. DNA polymerases contributed to 35% of the variation observed in spiked shellfish. A pairwise permutational multivariate analysis of variance (PERMANOVA) was performed and returned significant *P* values, <0.05. Spiked samples prepared with SuperScript II, SuperScript IV, or LunaScript RTs and AmpliTaq Gold DNA polymerase returned *P* values of 0.83 to 0.92, indicating a high similarity between obtained and expected sequences (Fig. S2).

RTs contributed to 37% of the variation observed in spiked shellfish. A *post hoc* test on the UniFrac distance matrix was performed using pairwise PERMANOVA with 999 permutations and returned adjusted *P* values of 0.84 to 0.93. This indicated high similarity between expected and obtained sequences when prepared with the same DNA polymerase (Fig. S3).

Custom BLASTn databases were used to assess the ability of the various protocols to return a 99% match to the previously obtained Sanger sequences. SuperScript II AmpliTaq Gold and SuperScript II Kapa Robust returned a 99% BLASTn match with bit scores of >500 and E values of <0.001 for all expected genotypes. SuperScript II Kapa HiFi I failed to produce a 99% match for the GI.9 genotype (Table S1).

Overall, LunaScript and AmpliTaq Gold provided the most accurate, high-quality results based on quality metrics, classification accuracy, and phylogenetic distance.

HTS amplicon sequencing of the capsid region permitted the detection of additional genotypes from naturally contaminated shellfish samples compared to the conventional Sanger sequencing.

Based on the preceding results, a library with naturally contaminated oysters was prepared using LunaScript in combination with AmpliTaq Gold for RT and the seminested PCR. Three naturally contaminated samples with various concentrations of norovirus GII were lysed and extracted for amplicon-based HTS and Sanger sequencing. Samples MIC15592, MIC16714, and MIC16945 contained 543 gc/g, 2,556 gc/g and 102 gc/g of norovirus GII, respectively.

Sequences obtained using Sanger sequencing could not be genotyped using NoroNet. However, CaliciNet and the internal classifier provided strong concordance of genotype assignment with the MiSeq results. Technical triplicates introduced from the first round of RT-PCR provided strong agreement regarding the relative abundance observed for each genotype detected (Fig. S4). More genotypes were detected using the amplicon HTS method than conventional Sanger sequencing of cloned variants in the naturally contaminated oysters. Three genotypes were detected using Sanger sequencing in MIC16714 (GII.6, GII.4 Sydney, and GII.13), while an additional genotype, GII.7, was detected using amplicon HTS. In MIC15592, GII.14 was detected using Sanger sequencing, while amplicon HTS detected both GII.14 and GII.6. Norovirus GII.6 was detected in MIC16945 using both Sanger and amplicon HTS.

## DISCUSSION

In this study, we demonstrated that reverse transcription and DNA polymerases impact HTS library quality. RT-qPCR data on the number of norovirus genome copies per gram was a moderate indicator of obtained HTS reads. The optimized extraction and seminested PCR method permitted the accurate detection of norovirus in naturally contaminated oysters when combined with a custom bioinformatic pipeline. This is an essential development for environmental virology. The application of HTS to genotype norovirus in contaminated foods has been constrained due to a lack of available methods.

This study focused on reverse transcription and seminested PCR steps for optimization. In terms of the quality of the sequences observed, as measured by Phred score and expected errors, combined enzyme choice shaped score profiles (Fig. 1 and 2). It has been well documented that the priming strategy and the RT can impact the reverse transcription of RNA to cDNA. However, previous norovirus HTS studies using custom hexamers reported no improvement in performance compared to random hexamers (8). While RT aims to produce cDNA that faithfully reflects the starting RNA sample, several studies indicate that the RT reaction can introduce large variability (32–34).

DNA polymerase contributed more to the variation observed for mean Phred scores, while RT contributed more to the variability observed for mean expected errors (Fig. 2). In particular, Kapa HiFi and KAPA Robust had higher expected errors when combined with LunaScript (Fig. 2B); conversely, AmpliTaq Gold and LunaScript provided the lowest expected errors overall. This implies that RT and DNA polymerase combinations operate synergistically. Nonetheless, the literature on the mechanism behind varying RT and DNA polymerase performance is limited. The initial publication describing AmpliTaq Gold outlined its superior performance in complex sample types with low genomic input and/or multiple PCR assays (35). At the same time, KAPA Robust has been recommended for amplification in samples with high levels of inhibitors (36). AmpliTaq Gold has been widely applied in molecular virology (37–39), though there is limited literature evaluating its performance relative to other DNA polymerases. In this case, LunaScript combined with AmpliTaq Gold provided the highest-quality sequences; however, previous studies with other complex sample types provide conflicting results (27, 29, 40–43). Several factors may contribute to the performance of RT and DNA polymerases, such as low-input genomic material, RNA quality, matrix-specific factors, target-specific factors, and DNA synthesis speed. PCR is a stochastic amplification process challenged by multiple templates, secondary structures, and GC content (30, 44). The predicted hairpin structures in norovirus (45, 46) and the presence of multiple genotypes in shellfish challenge the development of any HTS applications. This warrants further study, as it is important to understand why performance varies in order to optimize it.

Of note, AmpliTaq Gold provided high-quality sequences and optimal results in terms of classification accuracy when combined with specific RTs, even though the seminested PCR assay with AmpliTaq Gold was performed with the highest number of total cycles. The number of PCR cycles is known to influence results. While a higher number of PCR cycles might increase the likelihood that rare molecules are observed, it can also skew abundance estimates by amplifying the biases (29, 47). However, this was not the case in this study. There are no comparison studies on LunaScript, as it was only recently added to the market, but it is widely used for the RT step in the ARTIC severe acute respiratory syndrome coronavirus 2 (SARS-CoV-2) protocol (48). AmpliTaq Gold has a lower DNA synthesis rate than the other studied polymerases, Kapa HiFi and Kapa Robust. Furthermore, nanopore systems have demonstrated that lower translocation rates result in high accuracy (49); therefore, faster synthesis is not necessarily equally accurate, especially in the case of highly diverse amplicons.

Various attempts have been made to optimize the workflow in terms of the wet-lab methodology developed. Notably, applying the ISO 15216:2017 method in combination with the optimized seminested PCR did not successfully amplify norovirus in naturally contaminated samples. Several modifications were required, including the concentration of the viral RNA by eluting it into a lower volume and increasing the input cDNA in the first round of the seminested PCR. This emphasizes the importance of performing method development with the target matrix, in this case, naturally contaminated shellfish, as spiked shellfish samples performed well without modifications. The input concentration of viral RNA required for successful amplification in the seminested PCR was a key component of the method development. Naturally contaminated shellfish samples containing at least 100 gc/g of viral RNA as per the ISO 15216:2017 method could be successful sequenced if extracted with the modified protocol. This is line with previous studies in shellfish, specifying a minimum input concentration of 100 gc/g of norovirus RNA (16). In addition, the inclusion of technical triplicates incorporated in the various experiments resulted in improved results relative to instances where individual samples were used. This observation is consistent with previous work (8, 16).

Furthermore, it was notable that the pooling of amplicons from norovirus GI and GII PCR assays before library preparation resulted in lower classification accuracy, with markedly fewer reads aligning to norovirus GI. While these steps increase the workload per biological sample, we found that they are necessary for optimal HTS results. Enzyme choice (RT and DNA polymerase) did impact the accuracy of HTS of norovirus VP1 amplicons. Almost all combinations of enzymes returned perfect F scores (1.00) when used in experiments performed in triplicate, apart from those containing SuperScript II. Thus, all genotypes known to be present in the samples were detected with no false positives. In the first experiment, clinical samples and spiked shellfish samples were sequenced; the presence of GI.9 was not detected using SuperScript II in combination with Kapa HiFi in a clinical sample. Indeed, the GI.9 sequence in question has four known nucleotide mismatches with the primers used in this study. No genotypes were missed using the LunaScript/AmpliTaq Gold enzyme combination in spiked samples. The high concordance between MiSeq amplicon HTS and conventional Sanger sequencing results supports method application in naturally contaminated shellfish.

An important consideration in choosing suitable samples to process using the outlined methodology is the number of norovirus gc detected per gram, as per ISO 15216:2017 (50). Because of the moderate correlation between input gc per gram and obtained HTS reads, we advise selecting samples with more than 100 gc/g for norovirus amplicon HTS. Notwithstanding this recommendation, it was observed in this study that some samples with a high concentration of viral RNA may fail to produce peaks of the expected size (2100 Bioanalyzer), while samples containing <300 gc/g may produce high-quality sequences. This is likely due to the quality and fragmentation of the norovirus RNA present in the shellfish at hand.

The seminested PCR targets the VP1 capsid region of norovirus, and the RT-qPCR targets a smaller overlapping region in the VP1. Amplification or sequencing of the full-length VP1 region has been used as a proxy for infectivity based on the hypothesis that an intact capsid implies an intact virus capable of initiating an infection (51–54). As observed in this study, spiked samples may behave differently from naturally contaminated oysters, as the norovirus RNA is intact. In contrast, norovirus accumulated in shellfish could be degraded and fragmented by wastewater treatment processes (2, 55) and/or exposure to UV in the marine environment (56, 57). This supported the variation in the correlation between input material and obtained HTS reads from spiked and naturally contaminated samples (Table 3). Literature suggests this can partially be overcome by the choice of RNA extraction method, i.e., phenol-chloroform extraction with ethanol purification (12, 58), but yield is an important consideration in environmentally contaminated samples (58, 59). Despite this observation, bioaccumulation experiments with fragmented norovirus RNA and clinical samples established that the intact virus was preferentially bioaccumulated over fragments of viral RNA and could survive up to 2 weeks (60). In a previous trial, norovirus remained infectious for up to 61 days in groundwater at room temperature. It persisted for up to 3 years based on RNase^+^ RT-qPCR assays (61), though recent studies utilizing human intestinal enteroid (HIE) models have indicated a much shorter persistence of viable norovirus (62).

Furthermore, it has been well documented that noroviruses can be harbored within biofilms, resulting in increased persistence (63) and binding to histo-blood group antigen (HBGA)-like molecules on enteric bacteria, increasing persistence and enhancing viral pathogenesis (64, 65). As the target regions for RT-qPCR of norovirus are <100 bp, it is not surprising to observe a less-than-perfect agreement between the HTS reads for 340-/344-bp amplicons from a seminested PCR and number of gc per gram as per RT-qPCR amplicons. Therefore, it is challenging to define the probability that norovirus viral RNA detected by RT-qPCR in shellfish represents an intact and/or infectious virus (66, 67).

While this study builds on previous work (5, 8, 66) and enhances the capacity for surveillance and outbreak response on a national level, there are limitations. Much of the work presented was performed with spiked samples, which are not necessarily representative of naturally contaminated shellfish due to the quality and concentration of the RNA. Unfortunately, RNA quality is difficult to assess in molluscs due to a hidden break in the 28S rRNA (68), making it challenging to obtain an RNA integrity number (RIN). Therefore, we could not compare samples based on their RNA quality. Additionally, we could not represent the full genetic diversity of noroviruses due to limited access to clinical samples. Moreover, PCR-based sequencing will always be biased due to the choice of primers. Finally, while experiment design controlled for biological variation through the use of technical replicates across enzyme comparisons, variation introduced through library diversity and depth of coverage could not be excluded.

We hypothesize that updated primer sets would permit the detection of additional genotypes, particularly for norovirus genotypes GII.17, GII.3, and GI.3 (4, 20, 26, 69). While a confusion matrix was used to assess method performance, an interlaboratory ring trial would provide a more realistic measure of method performance. Our work has demonstrated the need for a modified RNA extraction protocol for amplicon HTS of norovirus in shellfish, and recent work indicates that RNA extraction modifications could improve metagenomics approaches (7). Future work should focus on addressing the impact of RNA concentration and purity on HTS results. Finally, clinical genotyping of norovirus relies on the RdRp and VP1 of norovirus (22, 70, 71). Ideally, the RdRp and VP1 should be amplified and sequenced in one amplicon, yet Illumina limitations concerning read length do not permit this. The application of Oxford Nanopore Technology or PacBio sequencing platforms would enable the sequencing of longer amplicons and merits investigation.

Studies in complex samples that employed amplicon HTS methods have demonstrated that amplicon HTS can characterize norovirus genotypes (5, 10, 16), but only two studies included validation of these results by Sanger sequencing (4, 6).

This study provides a fit-for-purpose protocol for the genotypic characterization of norovirus in shellfish. We determined that reverse transcription and DNA polymerase choice, technical triplicates, and an optimized RNA extraction procedure impact the quality and accuracy of HTS of norovirus amplicons. Wet-lab methodology optimization is pivotal in moving the field from *ad hoc* sequencing to accredited methods. The results provided here have wide-ranging implications for HTS study design. Establishing standardized and well-described HTS methods, from the wet lab to bioinformatic analysis, is vital for building consensus in outbreak investigations across shared jurisdictions. The methods we present here can be applied for widespread surveillance of norovirus in complex samples, such as shellfish or wastewater, to expand our understanding of norovirus diversity.

## MATERIALS AND METHODS

### Samples.

To assess the performance metrics, a series of three different experiments were performed.

First, a proof-of-concept library was prepared and sequenced using SuperScript II and a selection of different DNA polymerases in both clinical (Table 1) and spiked (Table 6) samples. A total of six stool samples were used as positive controls, four that were positive for genogroup II (GII.3, GII.2, GII.4 Sydney 2012, and GII.4) and two that were positive for genogroup I (GI.4 and GI.9), containing 1.67^3^ gc/μL to 3.23 × 10^6^ gc/μL. Twelve matrix-specific mock communities were prepared from these stool samples, with concentrations ranging from 597 to 14,292 gc/g (Table 6).

**TABLE 6:** Composition of matrix-specific mock communities or spiked samples sequenced using the amplicon HTS method in experiment 1

Community	Genogroup	Concn (gc/μL)	Presence of genotype						qPCR ABI (gc/g)^*a*^
			GI.4	GI.9	GII.3	GII.2	GII.4 Sydney	GII.4	
1	GII	2,000–5,000			Present	Present	Present	Present	13,424
2					Present	Present	Present	Present	14,292
3					Present	Present	Present	Present	13,705
4	GI	2,000–5,000	Present	Present					4,012
5	GII	1,000			Present	Present	Present	Present	2,868
6	Single genotypes	1,000	Present						1,233
7				Present					2,402
8					Present				700
9						Present			785
10							Present		1,209
11								Present	597
12	GI	1,000	Present	Present					4,279

a ABI, Applied Biosystems ABI 7500.

Spiked and naturally contaminated samples were prepared in triplicate for a comprehensive RT and DNA polymerase comparison in the second experiment. The spiked sample was obtained from the proficiency testing scheme operated by the European Reference Laboratory for foodborne viruses (sample MIC200561). MIC200561 contained approximately 10,000 copies of GI and 1,000 copies of GII (GI.3, GII.6, and GII.7), while MIC180026 was a sample from a harvesting site collected in January 2018. It contained approximately 1,000 gc/g GI and 4,500 gc/g GII.

In the final experiment, three naturally contaminated oysters harvested in 2015 and 2016 were subject to an optimized protocol (RNA extraction to RT-PCR) to demonstrate the application of the method in the target samples.

### Preparation of oyster and fecal samples for norovirus analysis.

In line with ISO 15216-1:2017, oysters were tested for the presence of norovirus GI and GII (72). In brief, oysters were cleaned before being shucked and dissected, with 10 oysters used per sample. The dissected digestive tissue (DT) was diced and combined was a sterile razor blade. Samples were lysed with 2 mL proteinase K (100 g mL^−1^), followed by incubation and shaking at 37°C for 60 min at 150 rpm. Samples underwent an additional incubation period of 15 min at 60°C. Supernatants were retained for RNA extraction following centrifugation at 3,000 × *g* for 5 min.

For each stool sample, 0.5 mL phosphate-buffered saline (PBS) (Oxoid, UK) was added to a 2-mL tube containing 2 g fecal material (neat) and vortexed vigorously. Then, 100 μL of the resuspended fecal material (neat) was transferred into a fresh tube containing 900 μL of PBS (10^−1^), and serial dilutions were prepared up to 10^−5^.

### Viral RNA extraction.

NucliSENS magnetic extraction reagents (bioMérieux) and the NucliSENS EasyMag extraction platform were used to extract RNA from 500 μL of DT supernatants. This was then eluted into 100 μL of elution buffer. RNA extracts were kept at −80°C until the RT-qPCR or seminested PCR analysis was carried out. A single negative extraction control (water) was performed.

### Determination of the norovirus concentration using one-step RT-qPCR.

For samples where quantification of the norovirus RNA is provided, RT-qPCR was performed per ISO 15216-1:2017 (72). RT-qPCR analysis was performed using the Applied Biosystems AB7500 instrument (Applied Biosystems, Foster City, CA) and the RNA Ultrasense one-step RT-qPCR system (Invitrogen). The following were combined on a 96-well optical reaction plate to prepare the reaction mixture: 5 μL of RNA and 20 μL of the reaction mix containing 500 nM forward primer, 900 nM reverse primer, 250 nM sequence-specific probe, 1× ROX (carboxyrhodamine) reference dye, and 1.25 μL of enzyme mix. Norovirus GI was detected using the previously described primers QNIF4 (73) and NV1LCR (74) and the TM9 probe (75), while QNIF2 (76), COG2R (77), and QNIFS probe (76) were used to detect norovirus GII. The internal process control mengovirus was detected using Mengo110, Mengo209, and Mengo147 probe (78). The plate was incubated at 55°C for 60 min and 95°C for 5 min, and then 45 cycles of PCR were performed, with 1 cycle consisting of 95°C for 15 s, 60°C for 1 min, and 65°C for 1 min. All samples were analyzed for norovirus GI and GII in duplicate. All control materials used in the RT-qPCR assays were prepared as previously described (79).

### Preparation of matrix-specific mock communities.

A panel of norovirus matrix-specific mock communities were generated using the positive-control material (Table 1). Based on the number of genome copies (gc) per microliter of each genotype, the weight of the norovirus-positive fecal sample to be added to the negative digestive tissue for the desired ratio was calculated and used to spike the homogenized norovirus-negative oyster digestive tissue. The total norovirus concentration in each mock community ranged from 597 gc/g to 14,292 gc/g of norovirus GI or GII RNA (Table 6).

### cDNA generation and seminested PCR.

cDNA was generated using either SuperScript II, SuperScript IV, or LunaScript RT as per the manufacturer’s protocols. Three DNA polymerases were evaluated for performance on the seminested PCR; AmpliTaq Gold, Kapa HiFi, and Kapa Robust. For AmpliTaq Gold, the first round of nested PCR was carried out with the following: 5 μL cDNA was added to a 45-μL reaction mixture with a final concentration of 10 mM Tris-HCl (pH 8.3), 50 mM KCl, 20 μM concentrations of deoxynucleoside triphosphates (dNTPs), a 2 μM concentration of each primer (Table 7), 2.5 mM MgCl_2_, and 2.5 U of AmpliTaq DNA polymerase (Applied Biosystems, USA). For the second round of PCR, the first-round PCR product (5 μL) was added to 45 μL of a reaction mixture containing 10 mM Tris-HCl (pH 8.3), 50 mM KCl, 20 μM dNTPs, 0.4 μM each primer, 2.5 mM MgCl_2_ and 2.5 U of AmpliTaq DNA polymerase.

**TABLE 7:** Seminested PCR primers targeting the norovirus VP1 capsid gene used to generate PCR products for Sanger and Illumina sequencing

Genogroup	Primer	Sequence (5′→3′)	Seminested PCR	Polarity	Reference
GI	COG1F	CGYTGGATGCGNTTYCATGA	1st	+	74
	G1SKR	CCAACCCARCCATTRTACA	1st and 2nd	−	77
	G1SKF	CTGCCCGAATTYGTAAATGA	2nd	−	77
	G1SKF NGS	GTCTCGTGGGCTCGGAGATGTGTATAAGAGACAGCCAACCCARCCATTRTACA	2nd, NGS	+	
	G1SKR NGS	TCGTCGGCAGCGTCAGATGTGTATAAGAGACAGCTGCCCGAATTYGTAAATGA	2nd, NGS	−	
GII	COG2F	CARGARBCNATGTTYAGRTGGATGAG	1st	+	74
	GIISKR	CCRCCNGCATRHCCRTTRTACAT	1st and 2nd	−	77
	GIISKF	CNTGGGAGGGCGATCGCAA	2nd	+	77
	GIISKF NGS	TCGTCGGCAGCGTCAGATGTGTATAAGAGACAGCNTGGGAGGGCGATCGCAA	2nd, NGS	+	
	GIISKR NGS	GTCTCGTGGGCTCGGAGATGTGTATAAGAGACAGCCRCCNGCATRHCCRTTRTACAT	2nd, NGS	−	

For the KAPA HiFi HotStart ReadyMix kit (Kapa Biosystems), the first round of PCR was carried out with the following: 5 μL cDNA with a 10 μM final concentration of primers, 12.5 μL of KAPA HiFi HotStart ReadyMix, and 2.5 μL of molecular grade biology water in a 25 μL reaction volume. For the second round of PCR, the first-round PCR product (2.5 μL) was added to 22.5 μL of a reaction mixture containing a final concentration of 10 μM for primers, 12.5 μL of KAPA HiFi HotStart ReadyMix, and 5 μL of molecular biology-grade water. For the KAPA2G Robust HotStart ReadyMix (Kapa Biosystems), the first-round PCR was carried out with the following: 5 μL cDNA with a 10 μM final concentration of primers, 12.5 μL of KAPA2G Robust HotStart ReadyMix, and 2.5 μL of molecular biology-grade water. For the second round of PCR, the first-round PCR product (1 μL) was added to 24 μL of a reaction mixture containing a 10 μM final concentration of for primers, 12.5 μL of KAPA2G Robust HotStart ReadyMix, and 6.5 μL of molecular biology-grade water. PCR conditions are described in Table 8.

**TABLE 8:** Seminested PCR conditions applied for the amplification of the norovirus VP1 capsid gene with selected DNA polymerases

Step	Conditions in indicated round for:					
	Kapa Robust		AmpliTaq Gold	Kapa HiFi	AmpliTaq Gold	
	1st	2nd	1st	2nd	1st (no heated lid)	2nd
Initial	95°C, 3 min	95°C, 3 min	95°C, 5 min	95°C, 3 min	95°C, 5 min	95°C, 5 min
Cycling	95°C, 15 s; 50°C, 15 s; 72°C, 15 s (40 cycles)	95°C, 15 s; 55°C, 15 s; 72°C, 15 s (30 cycles)	95°C, 1 min; 50°C, 1 min; 72°C, 2 min (40 cycles)	95°C, 30 s; 55°C, 30 s; 72°C, 30 s (25 cycles)	95°C, 1 min; 50°C, 1 min; 72°C, 2 min (40 cycles)	95°C, 1 min; 50°C, 1 min; 72°C, 2 min (40 cycles)
Extension	72°C, 1 min	72°C, 1 min	72°C, 15 min	72°C, 1 min	72°C, 15 min	72°C, 15 min

**FIG 3:**
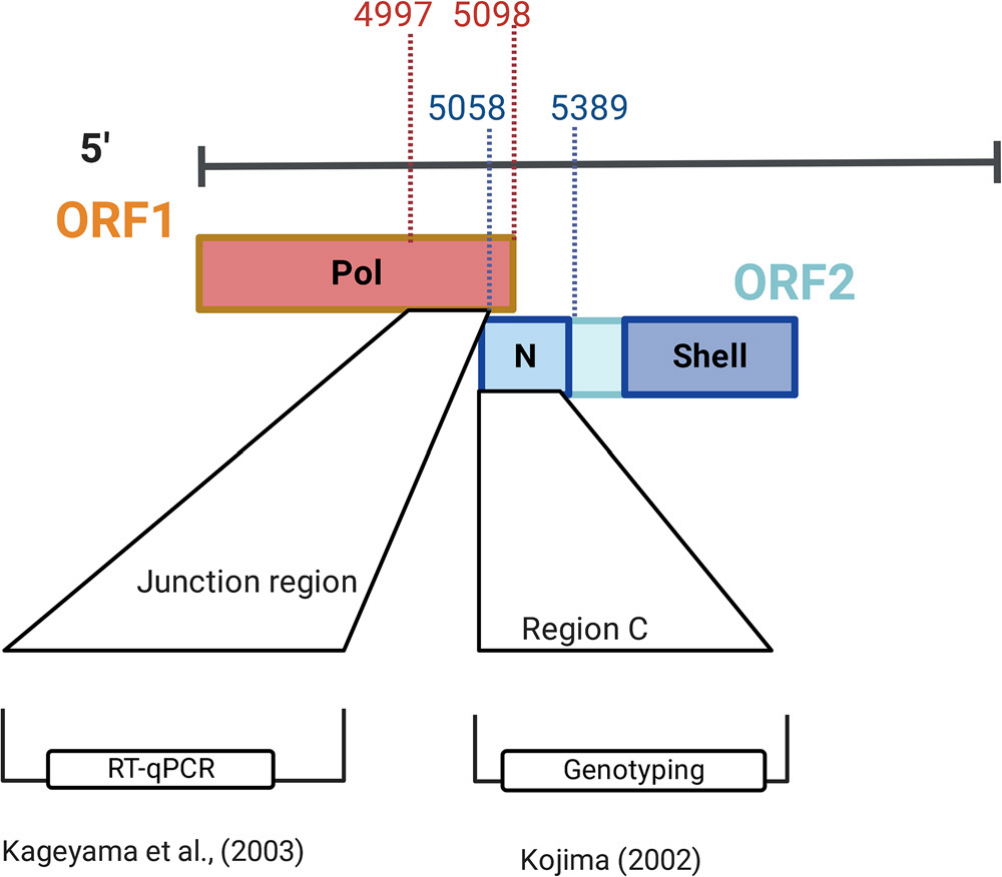
Regions of norovirus genome used for genotypic characterization and detection by RT-qPCR.

Individual or triplicate PCRs were performed during the first round of seminested PCR, using priming sites as shown in Fig. 3. The primers for Sanger sequencing and HTS (see NGS primers [listed in Table 7]) are provided in Table 7. Second-round PCR products were visualized on a 1× Tris-acetate-EDTA (TAE) 2% agarose gel containing 5 μL of ethidium bromide for clinical and spiked shellfish samples. The Agilent high-sensitivity DNA kit for naturally contaminated shellfish was used to visualize second-round PCR products for the Bioanalyzer 2100 system (Agilent Technologies).

### Cloning for Sanger sequencing.

PCR amplicons were gel extracted using the QIAquick gel extraction kit and ligated into the pGEM-T Easy plasmid (Promega). Vectors containing the PCR products were then cloned into chemically competent Escherichia coli. Diluted and undiluted cells were plated on Luria broth (LB) agar (Sigma, UK) containing X-Gal (5-bromo-4-chloro-3-indolyl-β-d-galactopyranoside; 20 mg/mL), IPTG (isopropyl-β-d-thiogalactopyranoside; 100 mM/mL), and ampicillin (100 mg/mL). Approximately 10 to 50 colonies per sample were picked and purified using the QIAprep spin miniprep kit. PCR then confirmed the presence of the target DNA with M13 forward (−20) (TGTAAAACGACGGCCAGT) and M13 reverse (−27) primers (CAGGAAACAGCTATGAC) with Kapa Robust as per the manufacturer’s instructions. Sanger sequencing was used to obtain the nucleic acid sequences of cloned fragments.

### MiSeq library preparation and sequencing.

Illumina sequencing adapters were incorporated into the second-round PCR primers; see Table 7 for the sequences of the primers. PCR products were purified using Ampure XP beads (Beckman Coulter) (0.8× bead-to-pool ratio) with elution of PCR products in 25 μL following the first bead cleanup. Cleaned-up PCR products were then indexed with the Nextera XT index kit (Illumina) following a modified 16S rRNA protocol from Illumina for use with the Nextera XT kit. Final indexed products (0.8× bead-to-pool ratio) were pooled to an equimolar concentration of 0.5 to 0.8 nM. The Agilent high-sensitivity DNA kit for the Bioanalyzer 2100 system (Agilent Technologies) was used to confirm amplicon presence, size, and adapter dimer removal. The cleaned pool was sequenced on an Illumina MiSeq sequencing platform with a 600-cycle V3 kit. All sequencing was performed at the Teagasc Sequencing Facility, per standard Illumina protocols.

### Optimized RNA extraction to seminested PCR method.

In order to improve the RNA yield from naturally contaminated shellfish, variations with regard to sample extract volume were included. RNA was extracted from 1,000 μL of proteinase K extract and eluted into a smaller volume (30 μL). All samples were extracted in duplicate. LunaScript and AmpliTaq Gold provided the highest-quality HTS reads with minimum errors. RT with LunaScript was performed in triplicate to provide sufficient cDNA for seminested PCR of norovirus GI and GII targets in triplicate. cDNA was pooled and stored at −20°C. The first round of seminested PCR was prepared as follows: 10 μL cDNA per technical triplicate was added to a 45-μL reaction mixture with a final concentration of 10 mM Tris-HCl (pH 8.3), 50 mM KCl, 20 μM dNTPs, a 2 μM concentration of each primer (Table 7), 2.5 mM MgCl_2_, and 2.5 U of AmpliTaq DNA polymerase (Applied Biosystems, USA). The first-round PCR product (5 μL) was subsequently added to 45 μL of a reaction mixture containing 10 mM Tris-HCl (pH 8.3), 50 mM KCl, 20 μM dNTPs, a 0.4 μM concentration of each primer, 2.5 mM MgCl_2_, and 2.5 U of AmpliTaq DNA polymerase. The Agilent high-sensitivity DNA kit for the Bioanalyzer 2100 system (Agilent Technologies) was used to visualize second-round PCR products. Following library preparation, an additional Ampure bead cleanup step (0.7× bead-to-pool ratio) was performed to remove adapter dimers, and 1 μL of the cleaned pool was visualized using the Agilent high-sensitivity DNA kit for the Bioanalyzer 2100 system (Agilent Technologies) to confirm adapter dimer removal.

### Bioinformatic analysis.

The pipeline utilized is based on the results from a previous study, which benchmarked pipelines and classifiers for norovirus amplicon analysis (80). Adapters and primers were trimmed using cutadapt (v 2.6) with a −E value of 0.1 and a minimum length of 100 bp. Reads were quality filtered in VSEARCH (v2.4.2) with a minimum length of 100 bp, a maximum length of 400 bp, a minimum overlap of 50 bp, a maximum of 20% mismatches in the alignment, and a maximum expected error threshold of 1. Chimera removal was performed using UCHIME within VSEARCH (v2.4.2) using *de novo* and reference-based chimera removal, with 99% clustering prior to chimera detection. The database for chimera-based removal was generated as follows: all available norovirus sequences greater than 1,000 bp were retrieved from GenBank using rentrez (v1.2.3). VP1 sequences were created using the second-round primers outlined in Table 7 in seqkit (v1.4), and sequences were clustered to 85% identity using CD-HIT (v4.7). Clustering of the sequences following chimera removal was performed at 99% identity, with a minimum of 1 read per sample required for a true sequence. Operational taxonomic units (OTUs) representing less than 1% of reads per sample were removed. OTUs were classified using the NoroNet typing tool from the National Institue for Public Health and the Environment (RIVM).

### Statistical analysis.

All analysis was performed using R statistics (v 4.2.1) in R Studio. Kruskal-Wallis tests were performed in base R, while the *post hoc* test for Kruskal-Wallis was conducted using the Dunn test in the R package rstatix (v 0.7.0) (81). Factor analysis of mixed data (FAMD) was performed using the R packages factoextra (v 1.7.0) (82) and factoMineR (v2.6.0) (83). DNA polymerase and RT were included as the factors of interest, alongside the numeric variable of interest, such as mean Phred score or mean expected errors. Kendall correlation tests were performed using R statistics and interpreted based on previously reported ranks (31).

Distance matrices were conducted using the R package vegan (v 2.6.2). All distance measures were conducted using 999 permutations (Jaccard). Analysis of variance using distance matrices (ADONIS2) was also performed using the vegan package in R with Bonferroni *P*-value correction. *Post hoc* tests for ANOSIM/ADONIS2 were performed using the R package RVAideMemoir (v 0.9-81-2).

A confusion matrix was generated using the yardstick package (v 1.0.9) in R from tidymodels (84). Data were coded in a binary fashion, using 1 for agreement between expected and observed data and 0 for disagreement. Classifiers and databases were compared based on the sensitivity or true-positive rate (TPR), the false-positive rate (FPR) or 1 − specificity, the F1 score, and balanced accuracy (average of sensitivity and specificity). Sensitivity refers to the probability of obtaining a positive test result for a true positive, and false-positivity rate refers to the probability of obtaining a false-positive test result for a true negative or, in this case, misclassification or missed classification. The F1 score (https://en.wikipedia.org/wiki/F-score) is the harmonic mean of the sensitivity and specificity, while balanced accuracy is the mean of the sensitivity and specificity. Jaccard distance measures (R package vegan v 2.6.4) were used to assess true and false matches between expected and observed data.

For UniFrac analysis, files were imported into QIIME2/2021.2. Sequences were aligned using the MAFFT plugin and masked. For UniFrac analysis, rooted trees were generated using rooted fastree and distances computed with all tips.

Custom BLAST databases were created based on the expected data for each library (1 and 2). Observed output for each sample was blasted against the custom database, requiring 99% similarity at 75% coverage of the amplicon. Multiple hits for an observed sequence to a reference sequence in the BLAST DB were filtered. The observed OTU with the highest bit score and lowest E value per sample/library was selected for the comparison if multiple hits were obtained.

### Data availability.

Sequence data generated during the current study have been deposited in the European Nucleotide Archive under accession number PRJEB58629. The scripts used for the processing of bioinformatic data are available at https://github.com/ahfitzpa/Norovirus_HTS_amplicon.
