# Supplementary material for: Amplicon-Based High-Throughput Sequencing Method for Genotypic Characterization of Norovirus in Oysters
Source: Appl Environ Microbiol. 2023 Apr 18;89(5):e02165-22. doi: 10.1128/aem.02165-22 (PMC10231197; doi:10.1128/aem.02165-22)

# Supplementary

## Jaccard supplementary for the section on how DNA polymerase influences the genotypic composition observed

Jaccard dissimilarity index was used to ascertain differences between the obtained genotypic compositions in the various enzymatic combinations. As evident in Figure 1. A and C, DNA polymerase had a moderate impact on the sequencing output, as supported by the moderate R^2^ value and significant p-value (0.001). A post hoc pairwise permutation MANOVA, as per Figure 1. D revealed that AmpliTaq Gold and Kapa HiFi were the most similar to the expected data, as per the adjusted p-values (Bonferroni correction). For RTase impact on genotypic composition, low R^2^ values were obtained from the Jaccard distance matrix in experiment 1; R^2^ 0.015-0.012. Nonetheless, an R^2^ value of 0.142 was obtained in experiment 2 with a p-value of 0.001. Therefore, DNA polymerase had a greater influence on the genotypic composition than RTase, with AmpliTaq Gold and Kapa HiFi performing similarly.


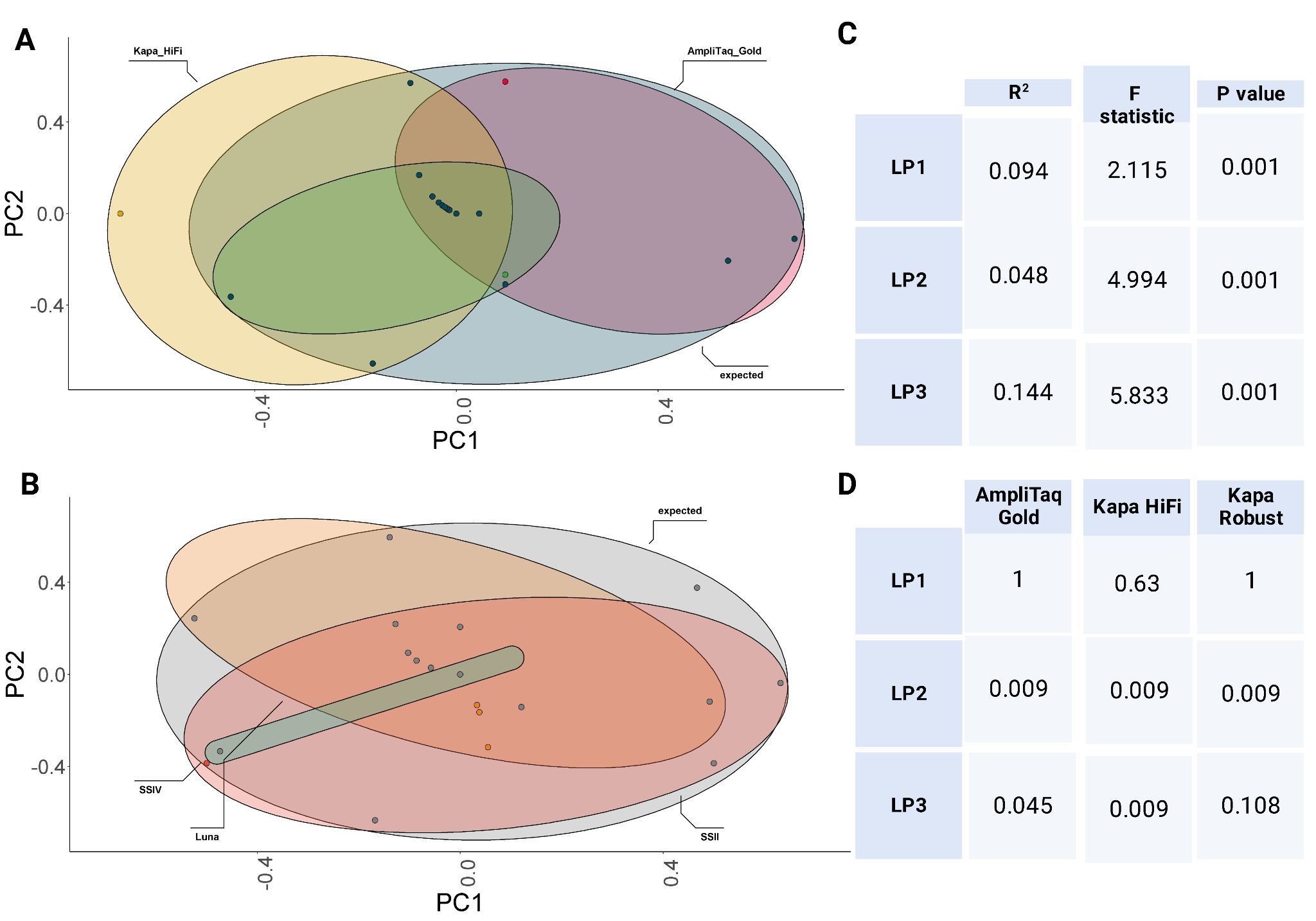


**Figure 1.** **A.** PCoA plot of Jaccard dissimilarity matrix based on presence/absence of expected vs sequenced genotypes in spiked oyster samples prepared with RTase enzyme SuperScript II (SSII) and three different DNA polymerases. AmpliTaq Gold generated amplicons with the greatest overlap with the expected genotypes. **B.** PCoA plot of Jaccard dissimilarity matrix based on presence/absence of expected vs sequenced genotypes in spiked oyster samples prepared with three different RTase enzymes and the DNA polymerase AmpliTaq Gold. LunaScript had the greatest overlap with expected sequences. **Analysis** of variance using Jaccard distance matrices reveals that the choice of DNA polymerase has a moderate impact on sequencing output, as supported by the R^2^ value and significant p-value (0.001). **D.** Pairwise permutation MANOVAs performed on the Jaccard matrix for DNA polymerases, revealed that AmpliTaq Gold and Kapa HiFi were the most similar to the expected data, as per the adjusted p-values (Bonferroni correction).

## UniFrac analysis

##
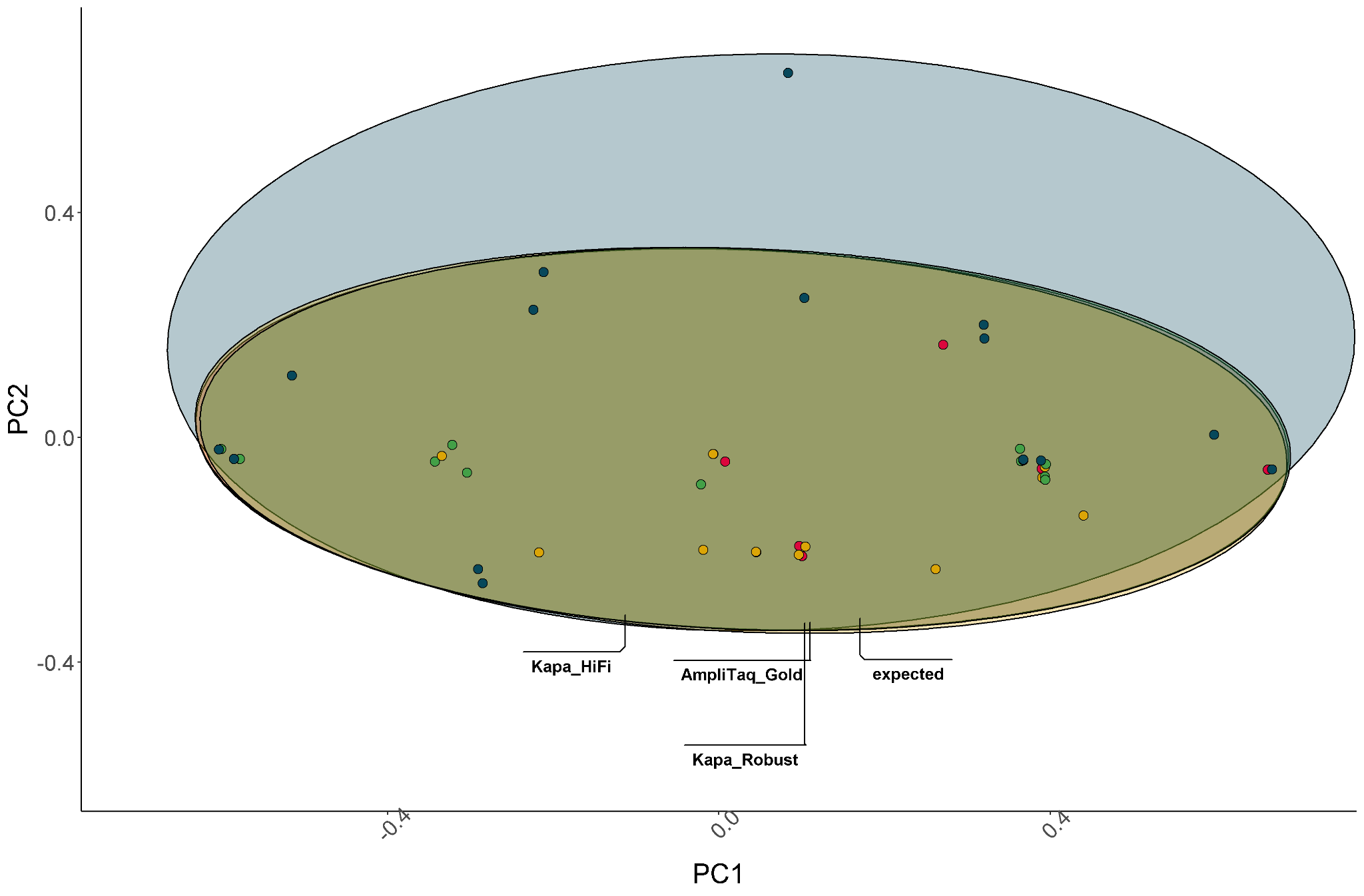


**Figure 2** PCoA plot of UniFrac index based on expected vs sequenced genotypes in spiked oyster samples prepared with RTase enzyme SuperScript II (SSII) and three different DNA polymerases. There was no significant difference between the DNA polymerases in terms of UniFrac distances, though they were significantly different to the expected sequences in experiment 1.


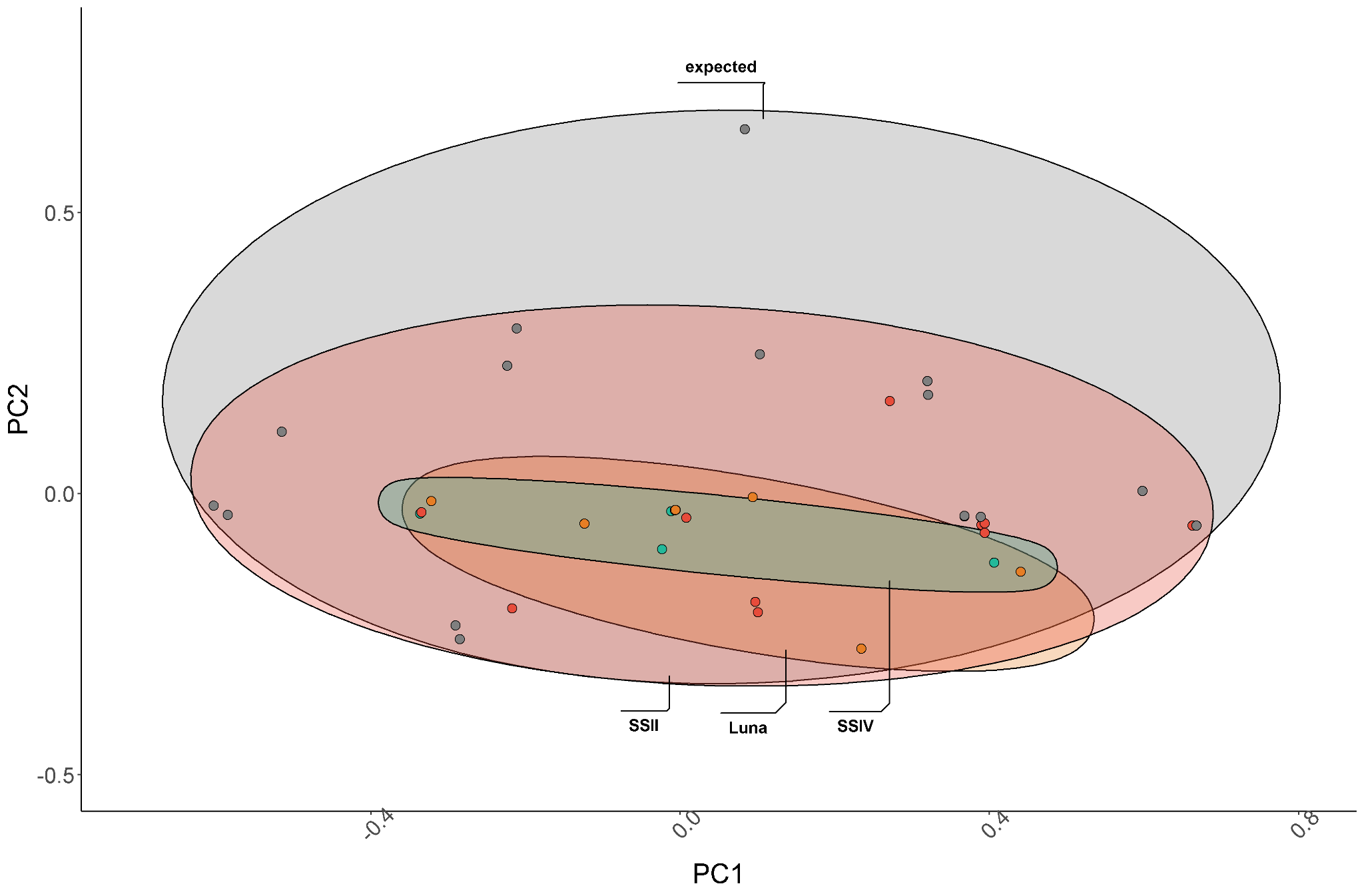


**Figure 3** PCoA plot of UniFrac index based on expected vs sequenced genotypes in spiked oyster samples prepared with AmpliTaq Gold and and three different RTases; SuperScript II, SuperScript IV and LunaScript. All RTases performed similarly.

## BLASTn of sequences obtained against Sanger sequencing

**Table 1.** BLASTn results of obtained HTS sequences in clinical and spiked shellfish compared to the Sanger sequences

| **Genogroup** | **Genotype** | **AmpliTaq Gold SuperScript II** | **Kapa HiFi SuperScript II** | **Kapa Robust SuperScript II** |
| --- | --- | --- | --- | --- |
| GI | GI.9 | 588 | ND | 588 |
| GII | GII.2 | 558 | 558 | 558 |
| GII | GII.3 | 558 | 558 | 558 |
| GII | GII.4 | 558 | 558 | 558 |
| GII | GII.4 | 558 | 558 | 558 |
| GII | GII.2 | 558 | 558 | 558 |
| GII | GII.3 | 558 | 558 | 558 |
| GII | GII.4 | 558 | 558 | 558 |
| GII | GII.4 | 558 | 558 | 558 |
| GI | GI.9 | 544 | 544 | 544 |
| GI | GI.4 | 538 | 538 | 538 |
| GI | GI.4 | 538 | 538 | 538 |

##

## Technical triplicates in LP4

**Figure 3** Relative abundance of genotypes detected in each sample by targeted norovirus amplicon HTS on MiSeq using the V3 kit. Technical triplicates demonstrate a strong concordance.
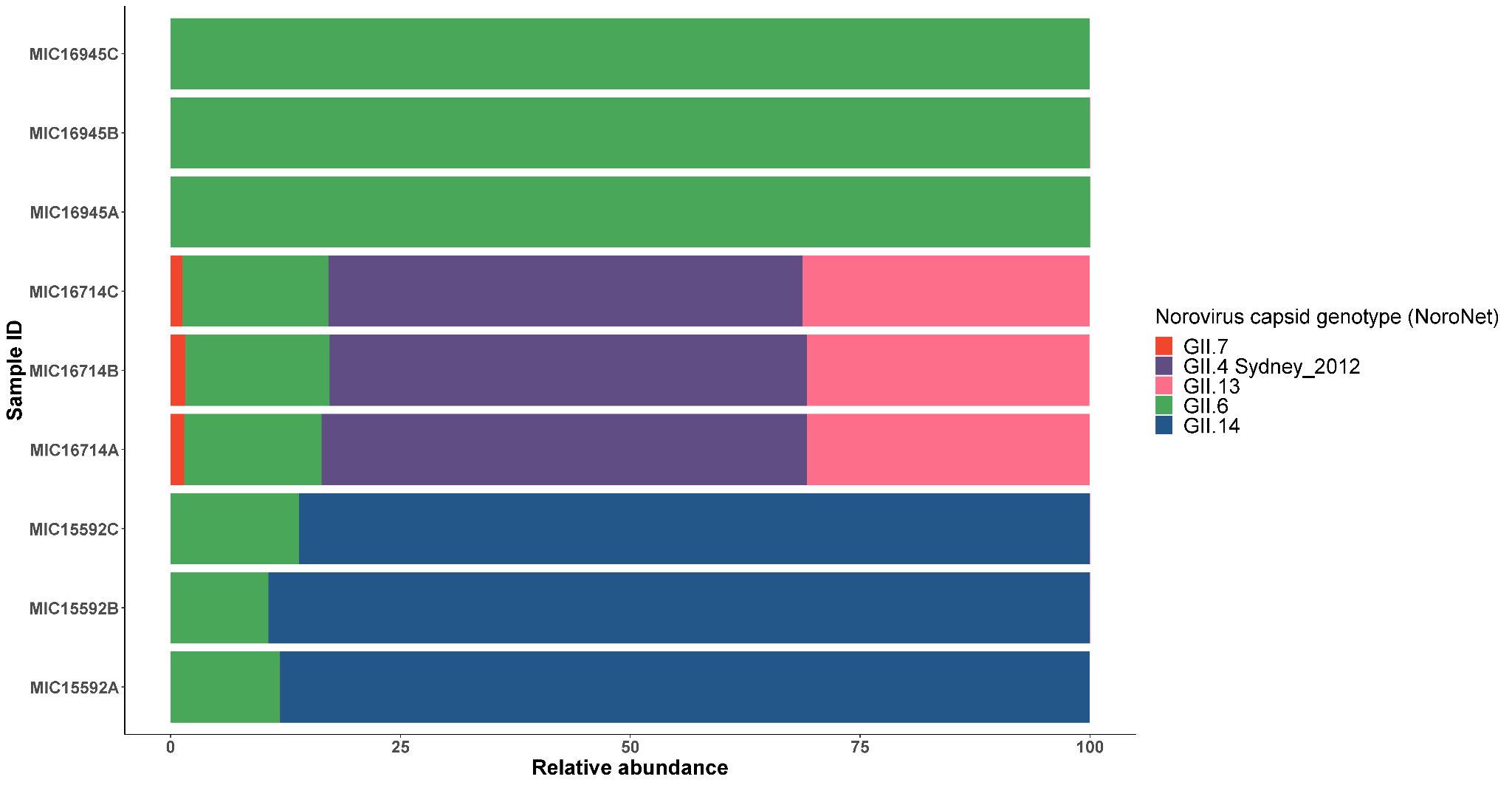

Supplement: Supplemental file 1 — Supplemental material. Download aem.02165-22-s0001.docx, DOCX file, 0.8 MB [file aem.02165-22-s0001.docx]
